# Electronic Consequences of the Formation of Local Tilting in Halide Perovskites

**DOI:** 10.1021/acs.jpclett.5c00814

**Published:** 2025-06-05

**Authors:** Young-Kwang Jung, Milos Dubajic, Samuel D. Stranks

**Affiliations:** † Department of Chemical Engineering and Biotechnology, 2152 University of Cambridge, Cambridge CB3 0AS, U.K.; ‡ Cavendish Laboratory, University of Cambridge, Cambridge CB3 0HE, U.K.

## Abstract

Complex structural dynamics in halide perovskites have attracted researchers in the field of perovskite optoelectronics. Although state-of-the-art experimental and theoretical techniques have disclosed the dynamic nature of local tilting in halide perovskites, how such local tilting influences the physical properties of these materials is still poorly understood. In this study, we model confined local tilting within a cubic halide perovskite CsPbBr_3_ and reveal the electronic consequences of the local tilting, employing density functional theory. Our calculations confirm that the locally tilted layers have larger band gaps than the host cubic lattice, where no charge trapping levels are introduced by them. The degree of local band gap fluctuation is predicted to depend significantly on the geometry of the locally tilted domains. We conclude that local tilting will act as dynamic charge-blocking barriers, which impact carrier dynamics in perovskite optoelectronic devices.

## Graphical abstract

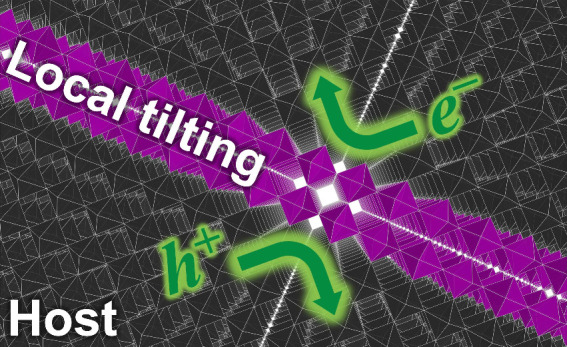

Structure–property relationships allow us to understand how the arrangement of atoms in a periodic unit cell is related to physical behavior of crystalline materials, ultimately helping us fine-tune atomic configurations in materials to enhance their performance in various applications.^1–4^ In the field of halide perovskite optoelectronics, it is well-known that the connectivity of octahedral networks governs optoelectronic properties of these materials and that corner-sharing networks are favorable for achieving high performance in photovoltaic and light-emitting devices.^5^ The corner-sharing octahedral networks have degrees of freedom for their tilting, which is a deviation from the ideal cubic symmetry and can significantly influence physical and chemical properties of halide perovskites including band gap^6–8^ and stability.^9–11^

To date, the structure–property relationships in halide perovskites have been understood based on static averaged tilting patterns throughout the entire crystal. However, recent works have reported dynamic local tilting patterns in these materials. Unlike global tilting, where entire octahedra in a cubic perovskite tilt toward a certain distortion mode, local tilting occurs only in several octahedral layers in the host cubic perovskite (Scheme 1). Via X-ray and neutron diffuse scattering techniques, dynamic formation and deformation of 2-dimensional (2D) “pancake-like” local tilting of octahedral networks were observed where the octahedra are following an out-of-phase tilting mode.^12–14^ A similar behavior of halide perovskites was also confirmed by molecular dynamics simulations based on classical and machine-learning interatomic potentials.^13,15,16^

**Scheme 1:**
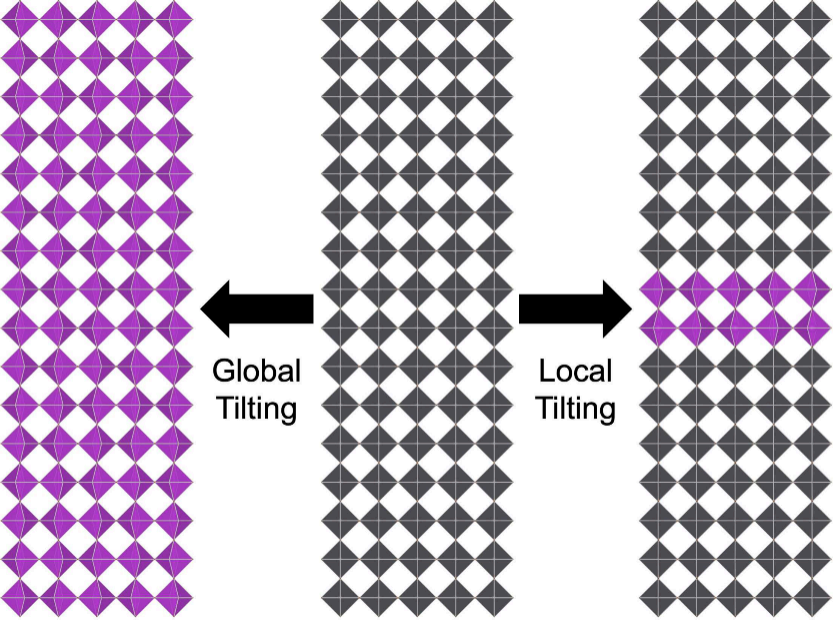
Illustration of Global Tilting and Local Tilting^a^

a The black octahedra represent untilted octahedra, whereas the purple octahedra represent tilted octahedra.

Although these recent studies have started to explore the possible electronic consequences of local tilting, a quantitative picture of how these local tiltings affect the electronic structure of halide perovskites is still lacking. Developing this understanding will be critical to appreciating the significance of such phenomena and exploring ways to employ them for enhanced performance and novel functionalities. Here, we explicitly modeled gradual formation of confined locally tilted layers in a cubic perovskite CsPbBr_3_ and performed first-principles simulations based on density functional theory (DFT) to understand electronic consequences of the formation of local tilting in halide perovskites. Our results demonstrate that locally tilted layers in the cubic perovskite act as charge blocking barriers where the geometry of the locally tilted layers determines the degree of barrier heights. This structural feature has important ramifications for charge carrier transport and recombination dynamics, suggesting new avenues to exploit or control these barriers in halide-perovskite-based optoelectronic devices.

In cubic CsPbBr_3_, the angle of Pb–Br–Pb bonds (θ_Pb–Br–Pb_) is 180°, whereas θ_Pb–Br–Pb_ in tetragonal CsPbBr_3_ is not 180° (Figure 1a,b). Here, we define the octahedral tilt angle as the difference between θ_Pb–Br–Pb_ in cubic and tetragonal CsPbBr_3_. By gradually distorting the octahedra in cubic CsPbBr_3_ supercells with an increment of 3°, we plotted double-well potentials of global and local tilting in Figure 1c,d (see the *Computational Details* section (in the last paragraph) for detailed explanation of our models). The minimum of the double-well potential was found at 27° for both global tilting and local tilting, but the well depth of global tilting (15.0 meV/atom) is found to be 20% deeper than that of local tilting (12.5 meV/atom). This indicates that the local tilting would exhibit a more dynamic nature than the global tilting as the dynamic hopping between wells requires less thermal energy when the double-well potential is shallower.

**Figure 1:**
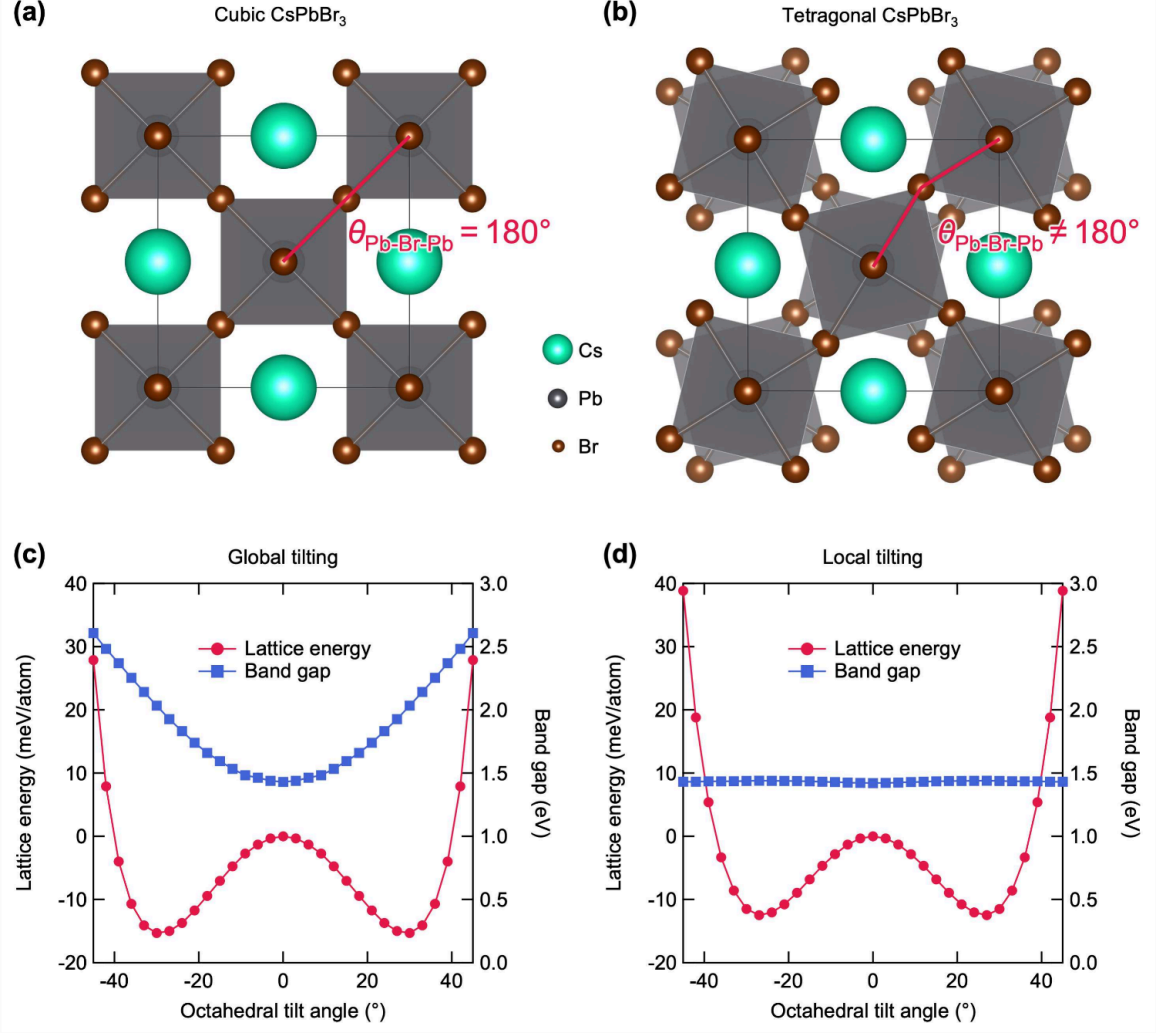
Top view of (a) cubic CsPbBr_3_ and (b) tetragonal CsPbBr_3_ with out-of-phase tilting. Double-well potential and change in band gap over gradual octahedral distortion for (c) global tilting and (d) local tilting.

Changes in the band gap of supercells in Figure 1c,d were obtained using the hybrid functional HSE06 ^17,18^ with a Hartree–Fock exchange (α) of 43%^19^ including spin–orbit coupling (SOC). In our calculation, the band gap of cubic CsPbBr_3_ (at a global octahedral tilt angle of 0°) is 1.43 eV, while the band gap of tetragonal CsPbBr_3_ (at a global octahedral tilt angle of 27°) increases to 1.93 eV. The increase in the band gap under global tilting is a well-known behavior of cubic halide perovskites.^20^ On the other hand, it is found that the band gap remains nearly constant at 1.42–1.44 eV when PbBr_6_ octahedral network locally distorts.

To understand this unique electronic behavior of local tilting, we further explored changes in electronic structures along with a gradual increase of the tilt angle. A $\sqrt{2}\times \sqrt{2}\times 16$ supercell of cubic CsPbBr_3_ containing the confined 2D “pancake-like” local tilting is shown in Figure 2a. Following our recent report,^14^ we set the innermost two octahedral layers in the supercell as locally tilted layers that exhibit out-of-phase tilting. To distinguish the electronic structure of locally tilted layers from that of the bulk, we analyzed the layer-projected density of states (DOS) for bulk layers, locally tilted layers, and the difference between them at given tilting angles (Figure 2b). At 0° local tilting, the DOS for both bulk layers and locally tilted layers are identical to each other. As the tilt angle increases, the contribution of locally tilted layers to the valence band maximum (VBM) and conduction band minimum (CBM) states decreases, while bulk layers remain constituting the VBM and CBM states. The difference in the DOS between bulk layers and locally tilted layers clearly highlights the separation of bulk dominant states at the band edges from local tilting states below VBM and beyond CBM within a single supercell. The noticeable changes in the DOS of the locally tilted layers at tilt angle greater than 42° can be attributed to significant Pb–Br bond elongation induced by the severe tilting, which reduces the Pb–Br orbital overlap.

**Figure 2:**
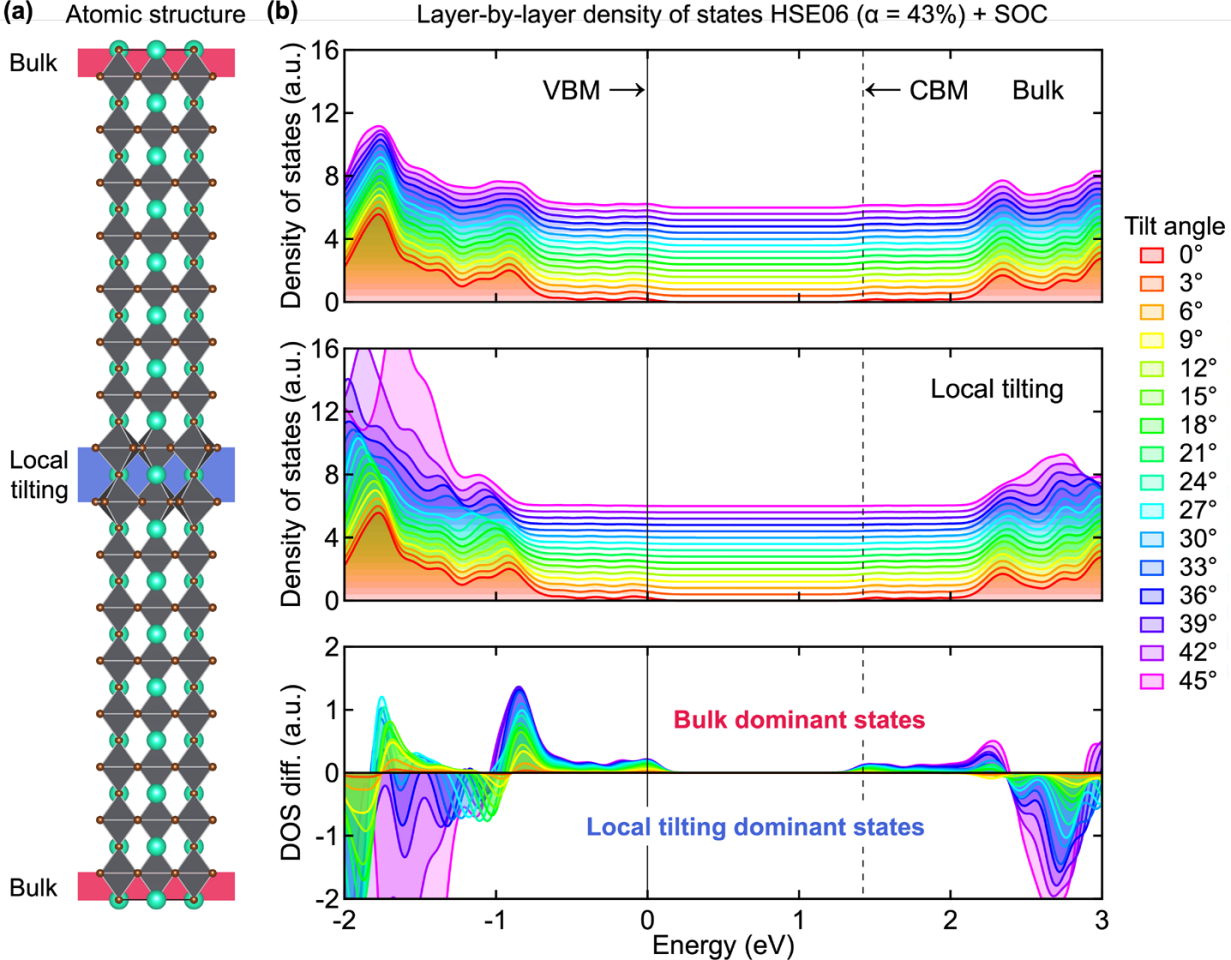
(a) Atomic structure of the CsPbBr_3_ supercell with local tilting. Bulk layers and locally tilted layers are represented as the red and blue regions, respectively. (b) Layer-projected density of states of bulk layers and locally tilted layers in supercells with local tilting as a function of tilt angle. HSE06 functional with a 43% of Hartree–Fock exchange including spin–orbit coupling was used.

As mentioned above, both CsPbBr_3_ and MAPbBr_3_ single crystals are known to have planar local tilting domains with out-of-phase tilting mode.^12,13^ However, in our recent report,^14^ FAPbBr_3_ single crystals were observed not to form planar local tilting domains but, rather, spherical local tilting domains, in which octahedral networks follow an in-phase octahedral tilting mode. In order to determine how different geometries of local tilting domains lead to different electronic consequences, we additionally modeled a cubic CsPbBr_3_ supercell with thick in-phase local tilting layers and compared its electronic structures with those of the cubic CsPbBr_3_ supercell with thin out-of-phase local tilting.

Figure 3a shows the atomic and electronic structures of a supercell with thin out-of-phase local tilting at a tilt angle of 27°. It is clearly shown that wave functions for both VBM and CBM states are localized in the cubic host layers, while no VBM and CBM wave functions are found in the locally tilted tetragonal layers. This further confirms that the cubic host layers maintain the band-edge states, as highlighted in Figure 2. To quantify the height of the blocking barrier, the eigenstates and density of states (DOS) of the supercells were further analyzed. By separating the contribution of the host layers and locally tilted layers to the eigenstates and total DOS, we confirmed the valence band offset (VBO) of 0.96 eV and conduction band offset (CBO) of 0.91 eV. In the case of thick in-phase local tilting (Figure 3b), wave functions for VBM and CBM are also localized in the cubic host layers, similar to the thin out-of-phase local tilting case. However, thick in-phase tilting layers exhibit a much smaller VBO of 0.40 eV and CBO of 0.34 eV. These results indicate that the local tilting domains would act as charge-blocking layers, effectively impeding both electron and hole transport.

**Figure 3:**
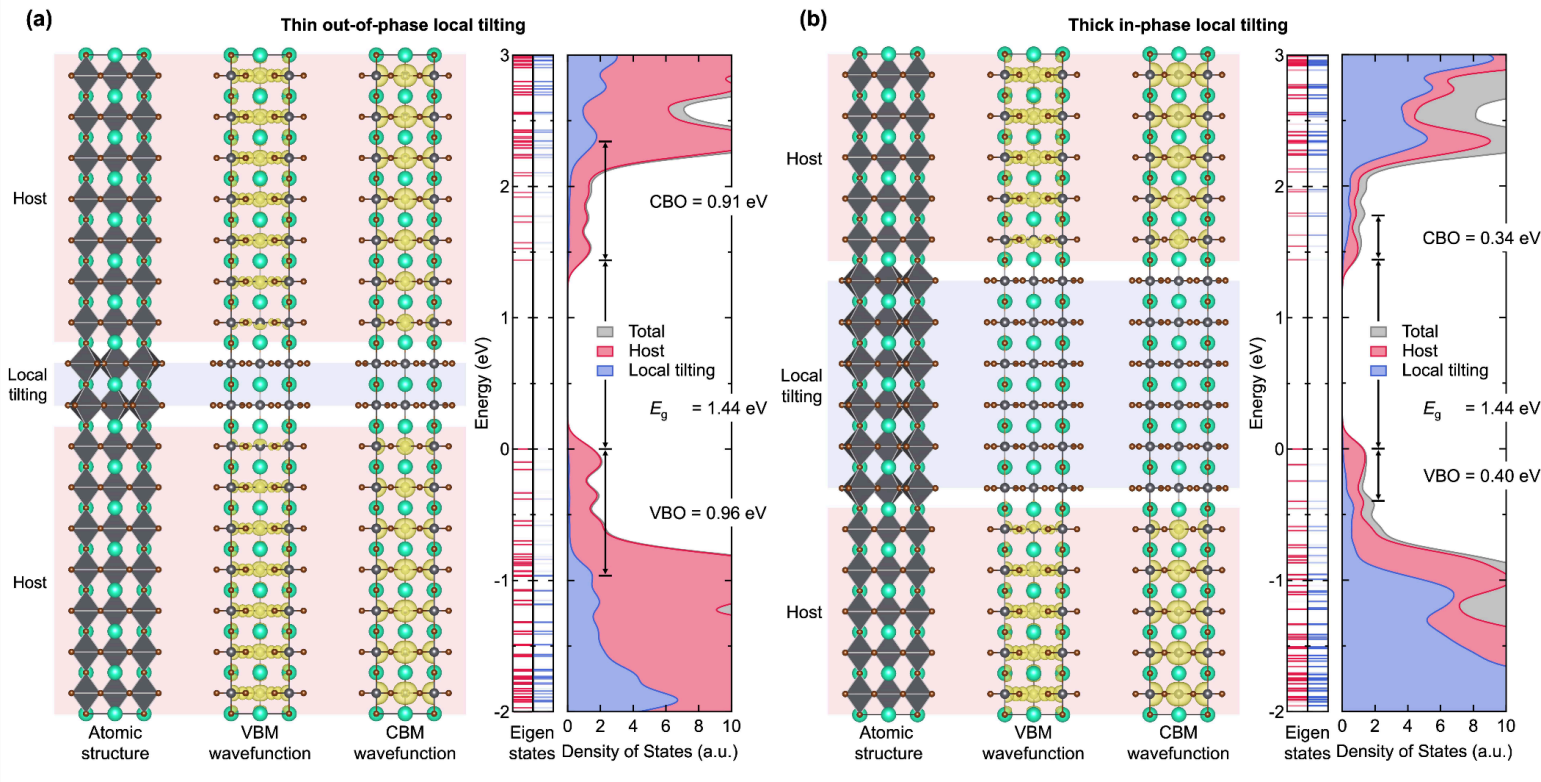
(a) Atomic structure of the CsPbBr_3_ supercell with thin out-of-phase local tilting, where wave functions of valence band maximum (VBM) and conduction band minimum (CBM) are plotted as yellow clouds. Red shades indicate host (cubic) layers while blue shades indicate locally tilted (tetragonal) layers. Calculated eigenstates and density of states for host layers and locally tilted layers are shown in red and blue, respectively. (b) Atomic structure, wave functions, eigenstates, and density of states of the CsPbBr_3_ supercell with thick in-phase local tilting.

Given that the difference in the band gap between cubic CsPbBr_3_ and tetragonal CsPbBr_3_ under global tilting is only 0.5 eV, the observation of such large VBO and CBO in locally tilted layers is surprising. We attribute this unique electronic property of the confined local tilting to a combination of physical phenomena including quantum confinement effects and splitting of degenerate VBM and CBM states, which arises from 2D-like thickness, reduced crystal symmetry, and lack of out-of-plane periodicity.^21–23^ The electrostatic potential is also found to be altered around the local tilting layers (see Figure S1). When locally tilted layers become thicker, the offsets decrease, suggesting that the increase in their thicknesses diminishes those effects.

We note that the tilting direction has little influence since both out-of-phase and in-phase tilting shows similar band gap deformation behavior.^12^ Hence, controlling the geometry of the local tilting domains is important for reducing the barrier height and optimizing the charge transport in halide perovskites.

Double-well potentials calculated for both thin out-of-phase and thick in-phase local tilting are plotted in Figure 4. The calculated double-well potentials were found to be almost identical to each other, indicating that PbBr_6_ octahedral networks themselves do not have any energetic bias on determining tilting direction^24^ and thickness of tilting layers (i.e., no correlation along the out-of-plane direction).^25^ These results therefore suggest that here what dictates the geometry of local tilting patterns in halide perovskites is the A-site cations. It has been reported that different A-site cations have a different nature of hydrogen bonding with octahedra^26^ and therefore closely couple with tilting behavior of octahedral networks in halide perovskites.^15,27^ In other words, a choice of the A-site cation can perturb the double-well potential of octahedral tilting, favoring a particular local tilting geometry. This alters the degree of intrinsic electronic disorder within a crystal and, consequently, leads to different optoelectronic properties of the materials.

**Figure 4:**
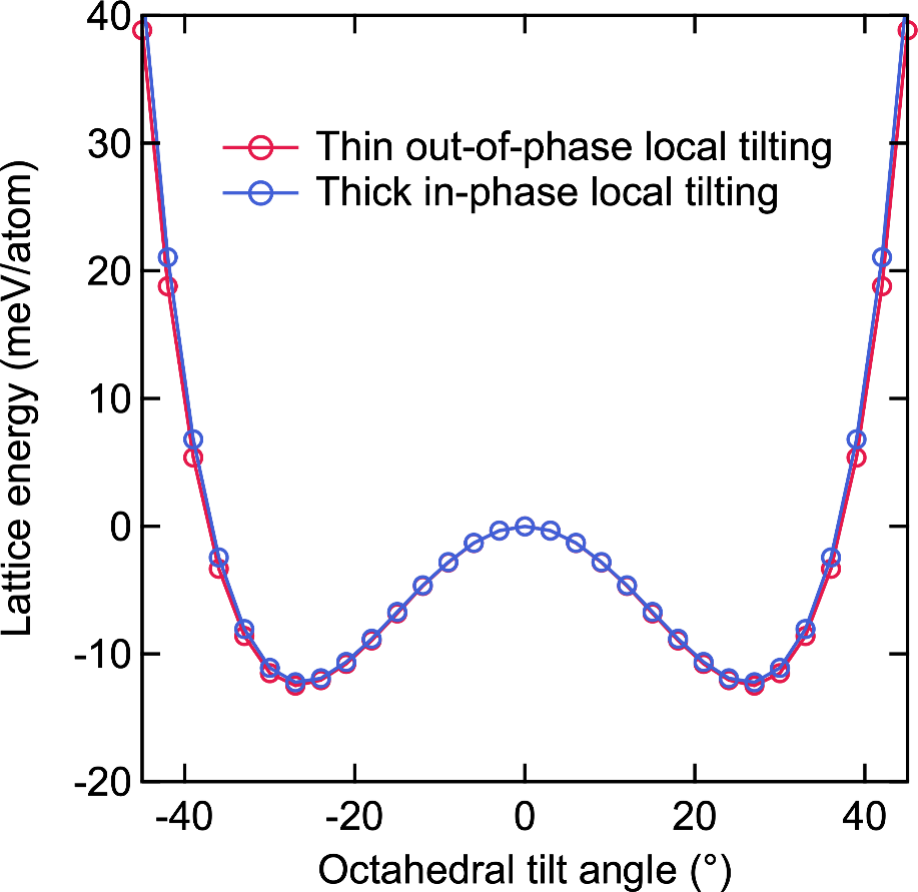
Double-well potential of thin out-of-phase local tilting and thick in-phase local tilting.

Experimentally, local tilting domains in MAPbBr_3_ were observed to have an average thickness of 6.3 Å and a diameter of 20.5 Å, while those in FAPbBr_3_ were observed to have an average thickness of 14.2 Å and a diameter of 21.5 Å.^14^ As a result of greater electronic disorder due to thinner (planar) local tilting domains in MAPbBr_3_ than FAPbBr_3_ that has smaller electronic disorder due to thicker (spherical) local tilting domains, MAPbBr_3_ single crystals were reported to have larger Urbach energies, wider full width at half-maximum (fwhm) of the photoluminescence (PL) peak, and lower photoluminescence quantum efficiency.^14^ Moreover, MAPbBr_3_ single crystals exhibit a shorter carrier diffusion length of 4.3 μm^28^ than FAPbBr_3_ single crystals (19.0 μm).^29^ These experimental observations collectively suggest the critical role of dynamic local tilting in dictating optoelectronic properties of halide perovskites, which can be mediated by the selection of A-site cations.

In summary, we investigated how the formation of local tilting domains influences the electronic structure of halide perovskites. Although describing such local domains from first-principles atomistic theories is challenging, we have modeled a practical computational system, a series of supercells that contain confined local tilting with infinite lateral extent, which offers a conceptual shift by distinguishing between global tilting and local tilting in halide perovskites. Our calculations have revealed that local tilting domains have a band gap larger than that of the cubic host and act as charge blocking layers. The degree of electronic disorder depends on the geometry of the local tilting domains, where A-site cations play a decisive role. The role of local tilting as a charge blocking barrier may seem akin to the role of hexagonal stacking faults in halide perovskites,^30^ yet a crucial difference lies in its dynamic nature; while stacking faults are static defects, local tilting domains can fluctuate over time. This transient nature would explain why such barriers can remain elusive in experimental observations and yet not unduly deteriorate macroscopic charge transport.

Our static model does not yet capture the full spatiotemporal evolution of local tilting, which may affect the effective height of the resulting charge blocking barrier. Nevertheless it establishes a quantitative link between local distortion and electronic band offsets, laying a foundation for future investigations aimed at a more refined description of these local structures. Further investigations that couple the local electronic disorder to (i) the real time- and length-scale dynamics,^31^ (ii) cation- and anion-alloying within the perovskite lattice,^32^ and (iii) presence of point and extended defects^33^ are warranted to fully elucidate their impact on device performance.

*Computational Methods.* All calculations were performed based on Kohn–Sham density-functional theory^34^ considering periodic boundary conditions. Vienna Ab Initio Simulation Package (VASP)^35,36^ was used where projector augmented-wave (PAW)^37,38^ pseudopotentials are implemented. The plane-wave kinetic energy cutoff was set to 300 eV, and the valence states of Cs, Pb, and Br were treated explicitly by 9­(5*s* ^2^5*p* ^6^6*s* ^1^), 14­(5*d* ^10^6*s* ^2^6*p* ^2^), and 7­(4*s* ^2^4*p* ^5^) electrons, respectively. For atomic structure optimization, the Perdew–Burke–Ernzerhof exchange-correlation functional revised for solids (PBEsol)^39^ was employed with convergence criteria of 10^–6^ eV and 10^–2^ eV/Å for total energy and forces on each atoms. During the structure optimization of super cells with gradual local and global tilting, all lattice vectors were allowed to relax, while all atoms’ x and y coordinates were fixed at their initial position. z coordinates of the atoms were allowed to relax. Changes in lattice parameters for supercells with both global and local tilting are tabulated in Table S1. For quantitatively accurate electronic structure calculations, we performed single-shot self-consistent calculation using a nonlocal hybrid functional (HSE06)^17,18^ with a Hartree–Fock mixing parameter α of 0.43 ^19^ including spin–orbit coupling (SOC) on PBEsol-optimized structures. To model global tilting, a $\sqrt{2}\times \sqrt{2}\times 2$ supercell expansion of the cubic CsPbBr_3_ unit cell (containing 20 atoms) was adopted, while a $\sqrt{2}\times \sqrt{2}\times 16$ supercell expansion (containing 160 atoms) was adopted to model local tilting. Γ-centered *k*-point grid of 6 × 6 × 4 and 6 × 6 × 1 were used for PBEsol structure optimization of the global tilting and local tilting supercells, respectively. Γ-centered *k*-point grid was then reduced to 3 × 3 × 2 and 3 × 3 × 1 for HSE06+SOC electronic structure calculations of the global tilting and local tilting supercells, respectively.

## Supplementary Material



## Data Availability

The raw calculation data of this study is available to download at the University of Cambridge Apollo Repository [https://doi.org/10.17863/CAM.118865].

## Notes

Electrostatic potential analysis and changes in lattice parameters under gradual formation of global and local tilting (PDF)
