# Supplementary material for: Electronic Consequences of the Formation of Local Tilting in Halide Perovskites
Source: J Phys Chem Lett. 2025 Jun 5;16(23):5862–7. doi: 10.1021/acs.jpclett.5c00814 (PMC12169654; doi:10.1021/acs.jpclett.5c00814)
Supplement: Supplementary file 1 [file jz5c00814_si_001.pdf]

Supporting Information

Electronic Consequences of the Formation of Local Tilting in Halide Perovskites

Young-Kwang Jung,[*,†] Milos Dubajic,[†] and Samuel D. Stranks[*,†,‡]

†*Department of Chemical Engineering and Biotechnology, University of Cambridge, Cambridge CB3 0AS, UK*

‡*Cavendish Laboratory, University of Cambridge, Cambridge CB3 0HE, UK*

E-mail: yj359@cam.ac.uk; sds65@cam.ac.uk

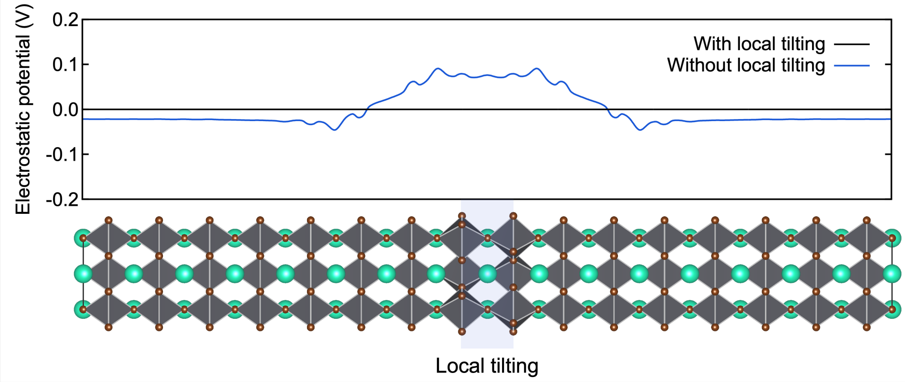

Figure S1: Macroscopic average of electrostatic potential of a supercell without local tilting and with local tilting.

Table S1: Changes in lattice parameters of supercells with global tilting and local tilting

	Global		Local (Thin)		Local (Thick)	
Angle (°)	a_0 (Å)	c_0 (Å)	a_0 (Å)	c_0 (Å)	a_0 (Å)	c_0 (Å)
0	8.288	11.720	8.288	93.763	8.288	93.763
3	8.288	11.720	8.288	93.763	8.288	93.763
6	8.287	11.721	8.288	93.763	8.288	93.763
9	8.267	11.728	8.288	93.763	8.288	93.763
12	8.259	11.733	8.284	93.790	8.277	93.828
15	8.242	11.754	8.282	93.808	8.270	93.855
18	8.220	11.762	8.280	93.810	8.263	93.907
21	8.195	11.785	8.277	93.817	8.254	93.966
24	8.165	11.822	8.274	93.837	8.244	94.040
27	8.131	11.852	8.270	93.866	8.233	94.133
30	8.092	11.907	8.267	93.885	8.220	94.238
33	8.050	11.962	8.262	93.915	8.207	94.371
36	8.004	12.029	8.259	93.936	8.195	94.490
39	7.960	12.102	8.255	93.982	8.182	94.642
42	7.915	12.191	8.252	94.004	8.173	94.822
45	7.886	12.275	8.250	94.023	8.165	94.983
